# Corticospinal neurons from motor and somatosensory cortices exhibit different temporal activity dynamics during motor learning

**DOI:** 10.3389/fnhum.2022.1043501

**Published:** 2022-11-25

**Authors:** Martín Macías, Verónica Lopez-Virgen, Rafael Olivares-Moreno, Gerardo Rojas-Piloni

**Affiliations:** Instituto de Neurobiología, Universidad Nacional Autónoma de México, Querétaro, Mexico

**Keywords:** motor learning, motor cortex, somatosensory cortex, fiber photometry, corticospinal tract

## Abstract

The ability to learn motor skills implicates an improvement in accuracy, speed and consistency of movements. Motor control is related to movement execution and involves corticospinal neurons (CSp), which are broadly distributed in layer 5B of the motor and somatosensory cortices. CSp neurons innervate the spinal cord and are functionally diverse. However, whether CSp activity differs between different cortical areas throughout motor learning has been poorly explored. Given the importance and interaction between primary motor (M1) and somatosensory (S1) cortices related to movement, we examined the functional roles of CSp neurons in both areas. We induced the expression of GCaMP7s calcium indicator to perform photometric calcium recordings from layer 5B CSp neurons simultaneously in M1 and S1 cortices and track their activity while adult mice learned and performed a cued lever-press task. We found that during early learning sessions, the population calcium activity of CSp neurons in both cortices during movement did not change significantly. In late learning sessions the peak amplitude and duration of calcium activity CSp neurons increased in both, M1 and S1 cortices. However, S1 and M1 CSp neurons display a different temporal dynamic during movements that occurred when animals learned the task; both M1 and S1 CSp neurons activate before movement initiation, however, M1 CSp neurons continue active during movement performance, reinforcing the idea of the diversity of the CSp system and suggesting that CSp neuron activity in M1 and S1 cortices throughout motor learning have different functional roles for sensorimotor integration.

## Introduction

The corticospinal (CSp) system, which originates from sensorimotor cortices (primary motor and somatosensory, M1 and S1, respectively) ending in the spinal cord (Lemon, 2008; Moreno-Lopez et al., 2016; Hooks, 2017), is maybe one of the most studied descending pathways. It plays a fundamental role in sensorimotor control and movement performance (Moreno-Lopez et al., 2016). Recent evidence shows that CSp neurons are segregated into different populations targeting specific zones and groups of interneurons in the spinal cord (Ueno et al., 2018). This suggests a modular organization of CSp outputs controlling sensorimotor behaviors in a coordinated manner. In this way, descending CSp projections drive the excitation and inhibition of motor neurons (Jankowska et al., 1976; Alstermark et al., 1992; Porter and Lemon, 1995) and modulate spinal reflexes by a variety of pre- and post-synaptic mechanisms (Evarts and Tanji, 1976; Wall and Lidierth, 1997). Additionally, CSp projections produce primary afferent depolarization and thus, presynaptic inhibition in cutaneous and muscular afferents (Eguibar et al., 1994). This indicates that the CSp system contributes to sensory information modulation and thus sensorimotor integration (Moreno-Lopez et al., 2016). CSp neurons of different cortical areas communicate each other in a coordinated yet differentiated manner for sensorimotor integration (Suter and Shepherd, 2015). But also, CSp neurons established bidirectional communication with fast-spiking and low-threshold-spiking interneurons (Tanaka et al., 2011; Apicella et al., 2012). This means that CSp excitability is dynamically regulated by intra and interareal cortical circuits.

The ability to learn motor skills (or “motor learning”) is characterized by an improvement in accuracy, speed and consistency of movements and involves multiple brain areas (Sanes and Donoghue, 2000; Dayan and Cohen, 2011; Li et al., 2017). The M1 cortex is particularly important as it is involved not only in movement execution but also in learning of new movements (Monfils et al., 2005; Papale and Hooks, 2018). This learning has been related to alterations in the dynamics of both, excitatory and inhibitory neuronal activity throughout the cortex (Makino et al., 2017) and the induction of learning-related plasticity, including changes in connection strength. In this way, learning-related plasticity has been reported in M1 cortical inhibitory circuits during motor learning, mainly in somatostatin-expressing (SOM) and parvalbumin-expressing (PV) inhibitory neurons. Particularly, PV neurons show a transient increase in the density of their axonal boutons during training, whereas SOM neurons exhibit a decrease in the density of their axonal boutons. Furthermore, activation or inactivation of only SOM neurons alters motor learning, leading to lower movement consistency and a decrease in the fraction of correct trials (Chen et al., 2015). Learning-related plasticity, like dendritic arborization and dendritic spine addition or elimination, has been also observed in CSp M1 neurons projecting to the contralateral forelimb-associated segments of the cervical spinal cord (Rioult-Pedotti et al., 1998; Wang et al., 2011; Papale and Hooks, 2018). The above highlights the importance of the interaction between excitatory and inhibitory cortical circuits for proper movement learning.

Individual M1 CSp neurons are related to movement; however, during motor learning, they can change their activity and they can switch between movement-related classifications throughout motor learning (Peters et al., 2017). Nevertheless, S1 CSp neurons in motor learning and execution have been poorly studied. CSp tract is anatomically and functionally segregated, controlling different spinal cord circuits responsible for modulating motor output and sensory input information in a coordinated manner (Olivares-Moreno et al., 2017, 2019). However, whether the corticospinal activity differs between cortical areas across motor learning remains unknown.

To further understand the functional organization of the CSp system and its roles in motor learning, we analyzed the simultaneous calcium activity using photometry of specific CSp neurons in S1 and M1 mouse cortices during the learning of a sensorimotor task involving planning and execution of a lever-press movement. We hypothesized that CSp neurons of different cortical areas (M1 and S1) play different roles during motor learning.

## Methods

### Animals

All procedures were carried out in strict accordance with the recommendations of the National Institutes of Health Guide for the Care and Use of Experimental Animals and Laboratory Animal Care [Official Mexican Standard (NOM) 062-ZOO-1999]. The procedures were approved by the local Animal Research Committee of the Instituto de Neurobiología at Universidad Nacional Autónoma de México (UNAM). We used six-week-old male or female C57BL/6 mice that were maintained at constant room temperature (22 ± 2°C) under a 12 h light/dark cycle.

#### Experimental design

The study was designed to explore the functional organization of CSp neurons in the mouse during the process of motor learning acquisition. Two different experiments were performed: First, the distribution of CSp neurons projecting to the cervical spinal cord was analyzed in the primary somatosensory and motor cortices using a neuronal retrograde tracer. In the second experiment, the activity of CSp neurons of these two cortical zones was analyzed and compared during a motor learning task involving movement preparation and execution.

### Retrograde tracer injections

To quantify the number of CSp neurons, we injected the retrograde neuronal tracer Fluorogold (FG) (Fluorochrome, LLC; 3% in distilled water) into the cervical spinal cord (*n* = 3 animals). Body temperature was maintained using a thermostatically regulated heating pad, and all surgical procedures were performed under sterile conditions. The animals were anesthetized with isoflurane/O2 gas (1.5%) and placed in a stereotaxic frame (World Precision Instruments, Inc., Sarasota, FL, United States, cat #E04008-005) and given an injection of 2% lidocaine (0.10 cc, s.c.) at the incision site. Then, a laminectomy was performed followed by an incision of the dura to expose cervical (C4–C5) spinal cord segments. FG was pressure injected (80-100 nl) using a Pico pump (WPI Inc., PV830 Pneumatic PicoPump) coupled to a calibrated glass injection capillary (BLAUBRAND ^®^ intraMARK REF 7087 07) at 600 μm lateral to the midline and 300 and 600 μm depth. After injection of the tracer, the incision site was thoroughly cleaned with saline and sutured. Five days after the injections, the mice were deeply anesthetized (pentobarbital 45 mg/kg i.p.) and transcardially perfused with 0.1 M phosphate buffer (PB) followed by 4% paraformaldehyde in 0.1 M PB. The brains were extracted and post-fixed overnight in 50 ml of paraformaldehyde. Then the brain was cut coronally in the sensorimotor cortex at 50 μm in an automated vibrating Microtome (Leica VT1200S, Deer Park, IL, United States). After, slices were mounted with SlowFade Gold (Molecular Probes, cat. num. S36937). The size of the injection sites was estimated automatically using ImageJ software (V 1.50i) outlining the periphery of the zone stained with the tracer in the center of each injection and computing the transversal area. For neuronal quantification, we only used experiments in which the injections were located within the gray matter of the spinal cord and injection sizes were equal.

Slices were double-immunolabeled to count retrogradely labeled cells (FG) with respect to all neurons (cells positive to neuronal nuclear protein NeuN). To do so, slices were selected and rinsed three times in 0.1 M PB, then permeabilized and blocked in 0.5% Triton X-100 (TX) (Sigma Aldrich #9036-19-5), 20% normal goat serum (NGS) (Jackson ImmunoResearch Laboratories #005-000-121) in 0.1 PB for 40 min at room temperature. Primary antibodies were diluted 1:500 (mouse anti-NeuN, EMD Millipore #MAB377) and 1:500 (Rabbit anti-FG, EMD Millipore #AB153-I) in 0.5% TX, 20% NGS in 0.1 M PB for 48 h at 4°C. Sections were rinsed three times with 0.1 M PB and incubated in secondary antibodies (1:500 goat anti-mouse IgG1 Alexa-647 and 1:500 goat anti-Rabbit Alexa-488 molecular probes, #A11008) for 3 h at room temperature in 0.1 M PB containing 20% NGS.

Mosaic images (resolution: 1.023 μm/pixel) of the sections containing the FG-labeled cells were obtained with a fluorescence microscope (Zeiss AXIO Imager.Z1) attached to a digital camera (AxioCam MRm, 1.3 MP) using the appropriate filters (FG: GFP for Alexa 488, NeuN: Rhodamine for Alexa 647) and acquired with a 10x objective (ZEISS Plan-APOCHROMAT, NA: 0.45). Additional detailed images (resolution: 0.66 μm/pixel) were acquired with a confocal microscope (Zeiss 780 LSM) using an objective LD PCI Plan-Apochromat 25x/0.8 Imm Korr DIC M27.

### Analysis of the retrograde tracers

ImageJ1.51u was used for the injection site quantification. The neurons were counted in Amira software (version 5.6) placing landmarks over each labeled neuron of the microscopy images aligned to magnetic resonance image (MRI) volume^1^ (Ullmann et al., 2015). To better visualize the spatial distribution of the labeled neurons, the mosaic images were superimposed with the MRI atlas of the mouse brain (Janke and Ullmann, 2015). This T2-weighted atlas has isometric resolution of 32 μm, which allows the visualization of para-sagittal slices similar to the mosaics. The histological mosaics were manually aligned with the MRI volume by selecting shared and clearly visible anatomical landmarks and using a linear transformation, as implemented in Amira. Once the mosaic images were aligned with the atlas, the positions of the CSp neuronal somas were labeled. In this way, a 3D map of the CS neurons was obtained. To compute relative neuron density, the soma distributions were obtained in 250 × 250 μm steps for the tangential plane, and vertical density profiles were computed in 50 μm steps along the vertical axes.

Then, the percentage of neurons in each area of the sensorimotor cortex (M1, M2, S1, and S2), was computed relative to all labeled neurons obtained per experiment. In this way, 3D representation map of the CSp neuronal density was obtained.

### Virus injection surgery

The activity of CSp neurons in M1 and S1 was analyzed simultaneously with photometry during the motor execution task. To do so, we infected the CSp neurons with a retrograde virus leading the expression of the genetically encoded calcium indicator GCaMP7s (pGP-AAVrg-syn-jGCaMP7s-WPRE; Addgene, Plasmid #104487-AAVrg). Injections (250–300 nl) were performed similarly to the retrograde tracer injections (see above), but in these animals, two optical fiber cannulas [Doric lenses, Québec City, Canada, cat # MCF_200/250-0.66_1 mm_ZF1.25(G)_FLT] were stereotaxically implanted in the left primary motor cortex (1.8 mm anterior; 1.3 mm lateral from bregma; 700 μm depth) and primary somatosensory cortex (0.0 mm anterior; 2.0 mm lateral from bregma; 700 μm depth) and secured to the skull with dental cement (C&B Metabond ^®^, Parkell, Edgewood, NY, United States).

### Behavioral task

Ten days before the training, the mice (*n* = 6 animals) were deprived of water (1 ml per day) and deprivation continues during all the training. A session consists of 30 min of training per day. On the first training day, the mice were placed in an operant conditioning box (18 × 16 × 15 cm) in which the lever was located 1 cm from the floor, the signal light at 7 cm and the water dispenser at 2.5 cm. The lever was located to the right to force the animals to press with the right forelimb. The elements of the operant conditioning box were controlled with a custom-made platform^2^. In the initial phase (five sessions), the mice were trained to press the lever to receive a drop of water (∼8 μl). In a next phase, the animals were trained to press the lever in response to a signal light. For this, the animals had to press the lever while the signal light remained ON (the maximum duration was 2 s). Immediately after lever pressing, the signal light switched OFF, a drop of reinforcer was delivered to the animal and a new trial started. The time interval between the trials varied randomly between 3 and 6 s. If the animals pressed the lever without the light, a 7 s time out would begin.

### Photometry

The animals were injected with the retrograde viral vector (pGP-AAVrg-syn-jGCaMP7s-WPRE) into the cervical spinal cord. Additionally, two optical fiber cannulas were implanted into contralateral S1 and M1 to simultaneously measure the bulk calcium activity of CSp neurons in both areas. Three weeks after virus injection and fiber implantation, the mice were deprived of water, and training started. The fluorescence emitted by CSp M1 and S1 neurons was detected simultaneously with a photometry system (Multi-Fiber Photometry System, PLEXON Inc., Dallas, TX, United States). The system uses a blue light (465 nm LED) reflected by a dichroic mirror to excite GCamP7s. The emitted GCaMP7s fluorescence was recorded (Excitation wavelength 470 nm, Emission wavelength 500–530 nm) with CineLyzer software (Version 4.3.0 PLEXON Inc.). The trajectory of lever pressings was recorded with a video camera (Integrated Imaging Solutions Inc. Model: FMVU-03MTC-CS). The sampling rate for the fluorescence emitted by CSp neurons, as well as the trajectory of the lever pressings recorded with a video camera were adjusted at 30 frames/s. Each trial lasted 5 s and started 400 ms before the cue (signal light). Forepaw movements for each trial (lever pressings) were analyzed offline with CineLyzer software (Version 4.3.0) and MATLAB R2020b (MathWorks, Inc.) to obtain the displacement during the time (Supplementary Movie 1).

Bulk fluorescence emitted by CSp neurons in M1 and S1 was recorded in the training sessions with the complete task (training with light), during all contralateral forelimb displacements (lever pressings) that occurred during individual sessions (session consists of 30 min of training per day). Custom routines written in MATLAB were used for the analysis. Fluorescence is expressed as ΔF/F, where F is the fluorescence intensity at any frame and ΔF is the difference between F and the resting fluorescence (minimum of the temporal average of raw fluorescence signal in a 3 s window). The fluorescence and simultaneous lever displacement data were aligned to the beginning of the signal light and the beginning of movement (when the lever reached 3 mm), and then all trials per session were averaged. The maximum fluorescence value (calcium peak amplitude) was measured in each trial of the four initial (Beginners) and last four sessions (Experts). Additionally, the latency between the beginning of the cue (light ON) and the mean peak of calcium activity (maximum of the averaged calcium signal of all the trials during a training session) was computed.

The changes in fluorescence were measured in each correct trial (Supplementary Movie 1) during all sessions. Changes in fluorescence were aligned to the beginning of a visual cue (signal light ON) and to movement initiation. To analyze the precise times in which calcium signal increase significantly for each type of CSp neuron (S1 CSp or M1 CSp), at each time bin (30 ms) we calculated the AUROC (Metz, 1978; Tan, 2009) to compare the distribution of fluorescence values for all the trials of basal activity (before cue) versus the rest of all fluorescence values.

### Statistical analyses

Statistical analyses were computed in MATLAB or Prism 8 (GraphPad). We applied the Shapiro-Wilk normality test to all data sets and then apply the adequate statistical test. To compare the CSp neuronal density between cortical areas, we used the One-way ANOVA test. To compare the reaction time, we used the Mann-Whitney test. To analyze movement performance, we computed Pearson’s correlation between all individual trajectories produced in all the sessions. Then, we compared the “r” correlation values between groups using the Mann-Whitney test. To compare the fluorescence values of basal activity versus the rest of all fluorescence values, we used the AUROC curve test. To compare the peak average calcium activity and the latency between the peak amplitude calcium activity and the beginning of a movement, we applied a Paired *t*-test, unpaired *t*-test, or Wilcoxon test accordingly to data distribution. Differences were considered significant starting at *p* < 0.05. Data are expressed as mean and standard deviation.

## Results

**FIGURE 1:**
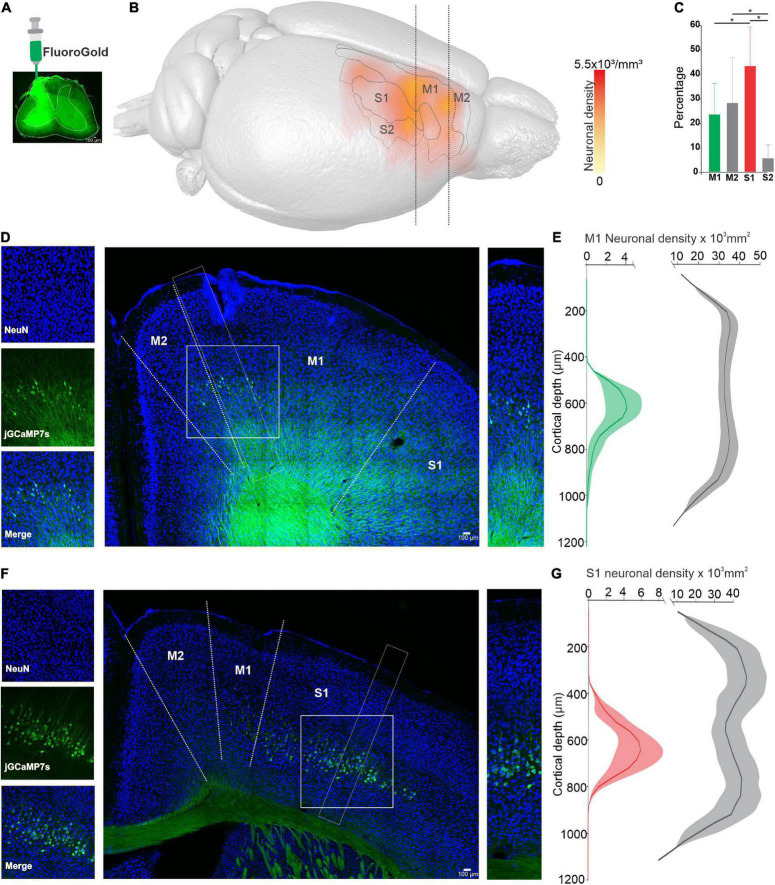
Distribution of corticospinal (CSp) neurons in sensorimotor cortical areas. **(A)**, Injection site of Fluorogold (FG) into cervical segments of the spinal cord (C4–C5). **(B)**, Average neuronal density map showing the distribution of CSp labeled neurons obtained from three animals. CSp labeled cells identified in the histological images were superimposed onto magnetic resonance image (MRI) volume (see methods). **(C)**, Percentage number of neurons (mean ± SD labeled in four areas of sensorimotor cortex (S1, M1, M2, and S2) defined by the MRI atlas. Significant differences are indicated with upper lines (*p < 0.05, One-way ANOVA). **(D)**, Coronal sections contralateral to the injection site showing CSp cells (green) and Neuronal Nuclear Antigen (NeuN) labeled cells (blue). Cropped images on the left and right show CSp and NeuN positive cells of the areas outlined by white dashed lines. **(E)**, Average density profiles computed in three consecutive slices for three experiments along the vertical axes of CSp neurons (mean green line, shadow SE) and NeuN labeled neurons (mean gray line, shadow SE). **(F,G)**, The same as **(D,E)** but for the S1 cortex. The scale of the cropped images **(D,F)** is the same as their respective neuronal density graphs.

First, we analyzed the detailed 3D distribution of CSp neurons in the sensorimotor cortex (see methods). The results show that CSp neurons projecting to the cervical spinal cord (Figure 1A) are located in areas corresponding to M1, M2, S1, and S2 (Figure 1B); however, the distribution in these four cortical areas is not homogeneous. The vast majority of CSp neurons are significantly denser in S1 (43.1 ± 16.1%; *p* = 0.03, One-way ANOVA) followed by M1 and M2 (M1: 23.3 ± 12.8%; M2: 28 ± 18.8%) and less dense in S2 (5.4 ± 5.6%) (Figure 1C). Specifically, S1 and M1 CSp neurons display a difference in the mean cortical depth where they are located (Figures 1D–G): CSp neurons in S1 are located 823.4 ± 138.5 (mean ± SD) μm below the pia, whereas in M1 they are at 849.4 ± 338.6 μm (*n* = 3 animals; *p* < 0.001, Mann–Whitney test).

**FIGURE 2:**
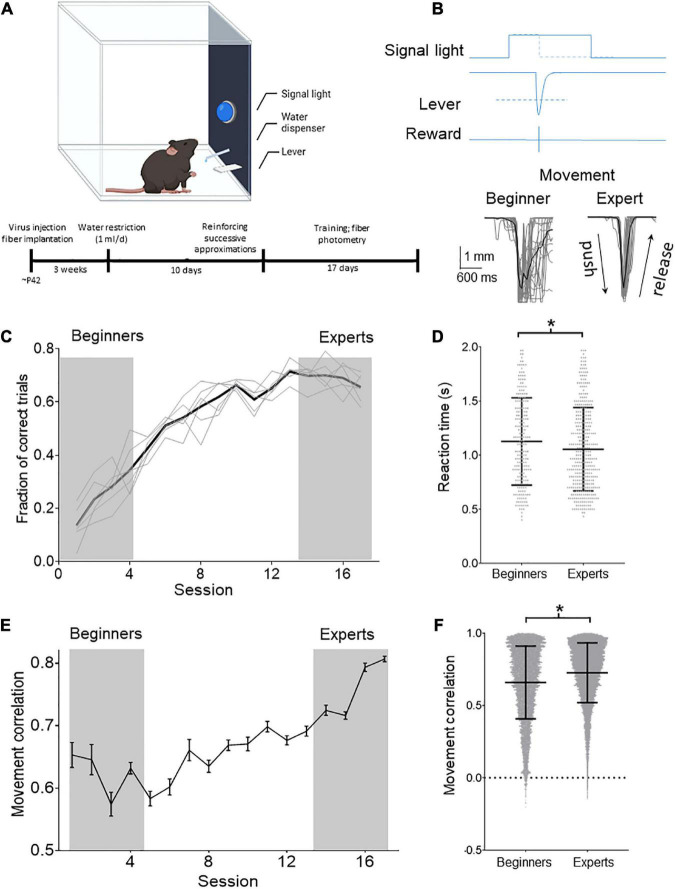
Operant learning in freely moving mice. **(A)** Schematic drawing of the task in which the animals learn to obtain the reinforcer in an operant conditioned manner. Below is shown the timeline with the experimental procedures. **(B)** Behavioral task: the animals learned to press the lever in response to a cue (signal light). The cue remained ON for 2 s, and the animals had to press the lever during this time to receive a reward (drop of water). When the lever reached a threshold (dashed line; 3 mm) to deliver the reward, the signal light was immediately turned OFF. The position of the lever was tracked with a video camera and digitized offline (gray traces). The lever positions of all the trials obtained in two sessions (one at the beginning of training and one when mice become experts) of one of the experimental animals are illustrated below (average black line). **(C)** Mean (black line) and individual temporal course (gray lines) of the proportion of correct lever pressings in relation to all the pressing performed by the animals per session. **(D)** Reaction times computed for individual trial computed for the first four (beginners) and last four training sessions (experts). Mean ± SD reaction times are shown. **(E)** Temporal course of Pearson’s correlation computed for all the trajectories performed by the animals during the session. **(F)** Distribution of movement trajectory correlations (lines indicate mean ± SD) computed for the first four (beginners) and last four training sessions (experts). *p < 0.05 Mann–Whitney test.

Previous results reinforce the idea that the CSp system is functionally diverse. Therefore, we tested if CSp neurons of distinct cortical zones of the sensorimotor cortex are modulated differentially during movements and if CSp neurons change their activity throughout motor learning. Thus, we trained animals using an operant conditioning paradigm (Figure 2A) to press a lever in response to a signal light (Figure 2B) to receive a reinforcer (water). The animals (*n* = 6) learned the task in approximately 17 sessions, reaching an efficacy (proportion of correct responses) of 65.68 ± 0.06% (Figure 2C). Movement performance was also gradually increased during the sessions, significantly reducing reaction time (Figure 2D) (6.4% experts respect to beginners; *p* < 0.05, Mann–Whitney test) and increasing the correlation between individual movement trajectories (Figure 2F) (10.1% experts respect to beginners *p* < 0.0001, Mann–Whitney test). Moreover, the analysis reveals that the variability (standard deviation) of the reaction time and correlation values decreases (4.4 and 17.6%, respectively) in the late sessions compared with training sessions (Figure 2F).

**FIGURE 3:**
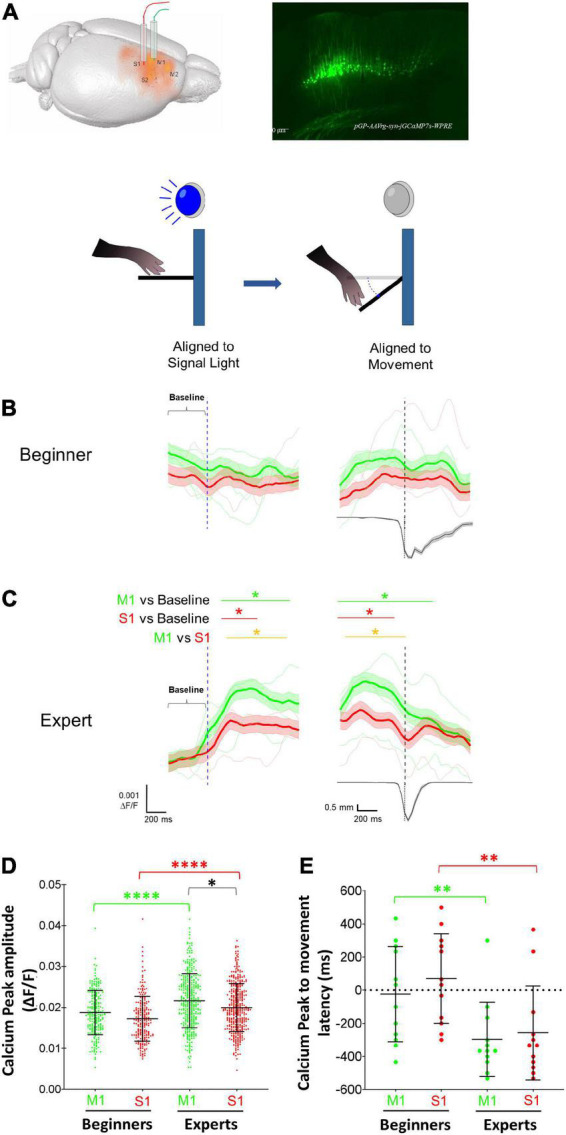
Modulation of primary motor (M1) and somatosensory (S1) corticospinal (CSp) neurons during the execution of forelimb movements. **(A)** Schematic diagram showing the location of the optical fibers into M1 and S1 cortices and the expression in CSp neurons of the GCaMP7s produced by the injection of the retrograde virus pGP-AAVrg-syn-jGCaMP7s-WPRE. Calcium activity photometry for CSp neurons was recorded in all trials of all learning sessions and the calcium fluorescent changes were aligned to the cue (light signal) and to the movement (threshold reached by the lever). **(B)** Grand average (mean: thick lines; SE: shaded areas) and individual mice averages (thin lines) of calcium fluorescence changes of M1 (green) and S1 (red) CSp neurons aligned to the signal light and movement (vertical dashed lines) computed for successful trials in the first four sessions (beginners). The lower trace is the averaged displacement of the lever computed for all the trials in the photometry sessions. **(C)** The same as **(B)** but photometry signals were recorded in the last four training sessions (experts). Horizontal green (M1 CSp) and red (S1 CSp) lines and asterisks above the traces indicate the time intervals in which fluorescence values increase significantly compared with baseline. Yellow line and asterisks indicates the time intervals in which M1 and S1 CSp fluorescent values distributions are significantly different between each other (*p < 0.05; AUROC curve test). **(D)** Distribution of peak amplitudes of calcium activity (mean ± SD) of M1 (green) and S1 (red) CSp neurons during movement epoch in each trial of the four initial sessions (Beginners) and last four training sessions (Experts). **(E)** Mean latency (± SD) computed per session between peak amplitude of calcium activity and the beginning of movement (see methods) of M1 (green) and S1 (red) CSp neurons during movement epoch in beginners and experts *p < 0.05; ^**^p < 0.01; ^****^p < 0.0001; Paired *t*-test, Wilcoxon test, Mann–Whitney test.

We used photometry (*n* = 3 mice) to analyze if calcium activity of CSp neurons in S1 and M1 cortices are modulated in a specific manner during a motor learning task involving movement preparation and execution (Figure 3A). The analysis reveals that in the early four sessions (beginners), calcium activity did not display a significant increment during cue alignment or movement alignment, indicating that CSp neurons do not synchronize their activity during the preparation and performance of movement in early sessions (Figure 3B). However, in the late four learning sessions (experts), a significant increase in calcium activity has been observed after the cue (signal light ON) (Figure 3C). Nevertheless, the temporal dynamics of calcium activity increments for CSp neurons located in M1 and S1 were significantly different (*p* < 0.05, AUROC curve test). S1 CSp neurons returned to the basal activity just before lever pressing started; in contrast, the increment of M1 CSp neurons was significantly longer, comprising lever pressing pushing and releasing lever phases (Figure 3C). From the calcium signal in M1 and S1 we compared the peak average activity (i.e., peak amplitude averaged across all trials in each session per animal) during movement epoch between beginners and experts. We found that the calcium signal peak amplitude increases significantly in both cortical regions (S1 11.7%; M1 16.6%) when the animals become experts but is significantly larger for M1 than S1 late sessions. (Figure 3D). Furthermore, we calculated the latency between the calcium signal peak amplitude and lever movement. This latency was time-locked in the beginners (S1 69.4 ms; M1 –25.1 ms), and negative (S1 –258.2 ms; M1 –296.9 ms), i.e., the peak amplitude in M1 and S1 preceded the lever movement, in experts (Figure 3E). No significant calcium increments were observed during omissions (trials without lever pressings). This indicates that a different temporal dynamic between M1 and S1 CSp neurons exists during the preparatory and movement performance phases.

## Discussion

Here, we describe in the mouse that CSp neurons projecting to the cervical spinal cord are anatomically broadly distributed in the contralateral cortex including motor (M1 and M2) and somatosensory (S1 and S2) cortices. It has been shown that different groups of CSp neurons project, in a segregated manner, to the same segment of the spinal cord in rats (Olivares-Moreno et al., 2017), mice (Asante and Martin, 2013; Kameda et al., 2019; Olivares-Moreno et al., 2019; Steward et al., 2021) and monkeys (Coulter and Jones, 1977). These studies show that CSp tract projections from M1 conspicuously avoid the dorsal horn, while S1 projections preferentially terminate into the dorsal horn. Additionally, we tested if the activity of CSp cells from M1 and S1 is different across motor learning by longitudinally recording the activity of CSp neurons from both cortices. We found that the population activity of both cortices did not change during the baseline epoch versus the movement epoch early in learning. Nevertheless, the CSp activity increased in amplitude and duration late in learning, displaying different temporal dynamics. Our results, together with previous reports (Olivares-Moreno et al., 2017, 2019) suggest that distinct populations of CSp neurons modulate different spinal cord circuits.

During training, we used water restriction as a reinforcer. It has been reported that properly hydrated animals with access to a tasty reward may take longer to learn a task, perform poorly, or even fail to perform it at all (Toth and Gardiner, 2000). Additionally, the consequences of water restriction appear to be minor. Performing health scores in mice, Guo et al. (2014) reported normal health conditions in mice continuously restricted for up to 4 months. In addition, Tucci et al. (2006) reported that weight loss due to water deprivation is less severe and more tolerable, making water deprivation less stressful for the animals, unlike food restriction. Moreover, when analyzing motor activity in the open field, no differences were found with respect to the control (see Tucci et al., 2006). In our study we have also monitored the weight of the animals to have an idea of the animal’s health and we adjust water consumption to maintain the mice with a body weight no more below 85% of their original weight throughout the training session (0.82 ± 0.04, mean ± SD), but enough to be motivated and able to perform the behavioral task, which is consistent with previous reports (Guo et al., 2014).

Mice learned the task and developed stereotyped movement; that is, more consistent and less variable movement. This is in line with the idea that variability can shape motor learning (see Dhawale et al., 2017). Early in training, variability is high and there may be motor biases unrelated to the task. However, as training progresses and reinforced behavior becomes more frequent, variability decreases and the sensorimotor system uses internal estimates to focus on relevant, reward-related information (see Dhawale et al., 2017). The fact that during training progress less variability in movement performance is accompanied by a significant increase in calcium activity of CSp neurons suggests that these neurons carry out an important function in learned movement performance. It has been reported that M1 is required for movement learning, but not for the execution of already learned movement (Kawai et al., 2015); however, it was not the case in our work. Furthermore, the more consistent learned movement is, the less engaged and required M1 is (Hwang et al., 2021). One hypothesis is that M1 is an exploration area with high variability, which makes it suitable for encoding learning of new movements. However, when the movement is well-learned, encoded information is sent to brain regions that may provide the automatized execution of the learned movement, as the dorsolateral striatum (Hwang et al., 2021; Wolff et al., 2022).

Primary motor is relevant for movement execution and motor learning. However, whether the activity of CSp cells from M1 and S1 is different across motor learning remains unknown. The changes in CSp activity were more prominent in M1 than in S1, denoting certain differences in their involvement during motor learning. The increase in the average population activity of CSp neurons during movement when mice are experts compared to the activity when they are beginners could be explained because: (1) the number of active neurons increases in the movement period; that is, more neurons are being recruited or (2) the activity of active neurons increases. Analyzing the involvement of M1 during long-term learning, Hwang et al. (2021) explored both alternatives by recording neurons in layer 2/3 of M1 while animals performed a two-direction joystick task. They found that the amplitude of activity was similar between different movements. However, when they classified neurons as active or not during each movement, they reported a higher fraction of activated neurons when mice performed one movement compared to another. Considering the findings above, our results suggest the occurrence of neuronal clusters in M1 and S1 that are temporally synchronized to encode the generation of the learned movement (Hwang et al., 2019).

The observation that population activity of M1 and S1 CSp neurons increases in parallel for some time during the task epoch may be related to the coding of movement characteristics. In studies with humans and non-human primates, it has been observed that reaching movement and its direction are parameters that may be encoded by neuronal populations from both motor and somatosensory areas (Georgopoulos et al., 1982, 1986; Prud’homme and Kalaska, 1994; Toxopeus et al., 2011; Chowdhury et al., 2020). It has not been shown that CSp cells directly encode the direction of movement and may participate indirectly in the encoding of reaching movement and its direction through information integrated by corticocortical and intracortical circuits (Papale and Hooks, 2018). Thus, CSp neurons integrate all this information to send dynamic output commands (Peters et al., 2017).

A significant finding from the current work is that, although the population activity of M1 and S1 CSp neurons increases in parallel for some time, M1 activity maintains the increase throughout lever movement while S1 does not. This dissimilarity between both cortical regions may be due to the coding of M1 related to force exerted by forelimb muscles. For example, Evarts (1968) found that M1 pyramidal neurons can change their firing rate in relation to force and its rate of change. Additionally, studies by Riehle and colleagues point to the fact that, within M1, there are independent neuronal populations whose activity may reflect aspects of movement such as reach and force (Riehle et al., 1994; Riehle and Requin, 1995; see Ashe, 1997). However, it is still unknown whether there is a relationship between S1 and the coding of the exerted force.

The idea of a functional segregation of neuronal populations within the same brain area has also been reported in the dorsolateral striatum (Sheng et al., 2019). Furthermore, shared dynamics has been reported in the activity of pyramidal neurons in premotor cortex and cerebellar granule cells, two anatomically distant but connected areas (Wagner et al., 2019). Historically, the CSp tract has been considered a unitary structure controlling motorneuron function. However, the results reported here reinforce that the CSp tract is organized into functionally and hierarchically organized sub-systems (Moreno-Lopez et al., 2016), controlling different spinal cord circuits in a coordinated manner. Overall, it is important to consider that motor learning may not be exclusively dependent on one brain region, but requires the involvement of multiple areas and neuronal circuits for suitable integration of input information and sending an appropriate output command.

The main limitation of our study is that the projection targets of the CSp neurons located in S1 and M1 cortices have not been identified, which is fundamental for understanding the mechanisms underlying the functional segregation of the CSp system. Nevertheless, the fact that different classes of spinal interneurons are targets of CSp projections (Ueno et al., 2018), indicates the functional relevance of CSp segregation for sensorimotor control. Consequently, one of the most important goals for the next studies is to analyze the target-specific intracortical circuits that allow CSp neurons to extract specific features from the same stimulus, which is relayed in parallel to the respective segmental targets controlling distinct neuronal circuits in a coordinated manner.

We conclude that different temporal activity dynamics of CSp neurons, located in S1 and M1 cortices, emerged during motor learning.

## Data availability statement

The raw data supporting the conclusions of this article will be made available by the authors, without undue reservation.

## Ethics statement

This animal study was reviewed and approved by Animal Research Committee of the Instituto de Neurobiología at Universidad Nacional Autónoma de México (UNAM).

## Author contributions

MM and VL-V carried out experiments. MM, VL-V, and RO-M performed the data analysis. MM and GR-P wrote the manuscript. GR-P conceived and designed the study. All authors contributed to the article and approved the submitted version.

## Footnotes

1
http://imaging.org.au/AMBMC/Model

2
https://github.com/Juriquilla-ENES-INB-A13/SkinnerDuino-shield
